# A review on mathematical models for estimating indoor radon concentrations

**DOI:** 10.1186/s40557-016-0091-6

**Published:** 2016-02-25

**Authors:** Ji Hyun Park, Dae Ryong Kang, Jinheum Kim

**Affiliations:** Department of Mathematics, Ajou University, Suwon, 16499 South Korea; Department of Humanities and Social Medicine, Ajou University School of Medicine, Suwon, 16499 South Korea; Department of Applied Statistics, University of Suwon, Hwaseong, 18323 South Korea

**Keywords:** Mathematical model, Indoor concentration, Radon entry, Exposure

## Abstract

Radiation from natural sources is one of causes of the environmental diseases. Radon is the leading environmental cause of lung cancer next to smoking. To investigate the relationship between indoor radon concentrations and lung cancer, researchers must be able to estimate an individual’s cumulative level of indoor radon exposure and to do so, one must first be able to assess indoor radon concentrations. In this article, we outline factors affecting indoor radon concentrations and review related mathematical models based on the mass balance equation and the differential equations. Furthermore, we suggest the necessities of applying time-dependent functions for indoor radon concentrations and developing stochastic models.

## Background

Exposure to radiation from natural sources leads to various environmental diseases. Radon, which is the primary constituent of natural radiation [1], is the leading environmental cause of lung cancer next to smoking [2]. Studies in Europe, the United States, Canada, and China have effectively shown a relationship between indoor radon concentrations and lung cancer [3].

**Fig. 1:**
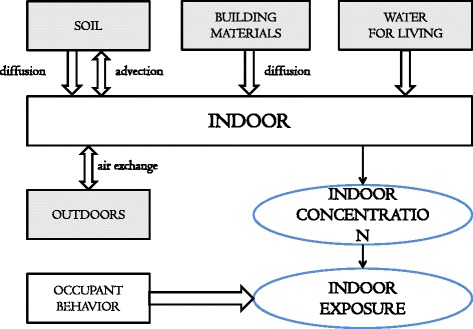
Diagram of factors governing indoor radon concentrations and exposure^a^. ^a^This diagram was modified from Font [5] and Font and Baixeras [6]. Radon in the soil permeates a building: diffusive and advective flow; radon from BM diffuses into buildings. A building’s water supply and natural gas are also sources that introduce radon into a building, although that from natural gas is not generally considered because it is difficult to measure and contributes to only very small concentrations. Additionally, indoor radon concentrations can also vary as a result of air exchange between indoor and outdoor air. Therefore, a subject’s indoor radon exposure in a building can be mainly determined by his or her behaviour and indoor radon concentration of the building that he or she resides

To establish a relationship between indoor radon concentrations and lung cancer, estimating an individual’s cumulative indoor level of radon exposure is necessary. For a period of time, *t*_1_ to *t*_2_, an individual’s cumulative level of indoor radon exposure can be defined as

$$ V{\displaystyle \underset{t_1}{\overset{t_2}{\int }}}{C}_i(t)dt, $$

where *C*_*i*_(*t*) is the indoor radon concentration (Bq/m^3^) at time *t* and *V* is the volume of the building of interest (m^3^). *C*_*i*_(*t*) can be affected by several factors: The United Nations Scientific Committee on the Effects of Atomic Radiation (UNSCEAR) reported that the 56 % of the radon that enters into a building comes from the soil, 21 % from building material (BM), 20 % from outdoor air, 2 % from a building’s water supply, and 1 % from natural gas [4]. As shown in Fig. 1 (see [5, 6] for more details), radon in the soil permeates a building: diffusive and advective flow; radon from BM diffuses into buildings. A building’s water supply and natural gas are also sources that introduce radon into a building, although that from natural gas is not generally considered because it is difficult to measure and contributes to only very small concentrations. Additionally, indoor radon concentrations can also vary as a result of air exchange between indoor and outdoor air.

There had been several studies to estimate the indoor radon concentrations with mathematical models. Ramola et al. [7] estimated the indoor radon concentration using a mathematical model based on the radon flux in soil and groundwater. Arvela et al. [8] calculated the indoor radon concentrations with a mathematical model which considers diffusion from soil and BM sources and air exchange simultaneously. These calculations have been compared with the measured variations of Finnish dwellings. Font [5] and Font and Baixeras [6] developed a generic and dynamic model of Radon Generation, Entry, and Accumulation indoors (RAGENA) which describes all the known sources including soil, BM, and water, and this model has been adapted to a Mediterranean climate house and Swedish house [9, 10]. Mathematical models for estimating indoor radon concentrations in high-rise buildings have been conducted by Man and Yeung [11] and Shaikh et al. [12]. These models included the contributions from BM and outdoor air. Moreover, model of indoor radon concentration in thermal spas was developed by Vogiannis and Nikolopoulos [13].

Most of the mathematical models reviewed in this article are based on the mass balance equation and differential equations. They can all be solved by numerical methods. The notation included in the mathematical equations reviewed in this article is listed in Table 1.

**Table 1:** Notation

Symbol	Unit	Description
*C*_*bm*_	Bq/m^3^	Radon concentration in building material (BM)
*C*_*i*_	Bq/m^3^	Indoor radon concentration
*C*_*o*_	Bq/m^3^	Radon concentration of outdoor air
*C*_*s*_	Bq/m^3^	Radon concentration in soil
*C*_*w*_	Bq/m^3^	Radon concentration in water supply
*F*_*s*_	Bq/(m^2^ · s)	Total radon flux from soil into building
*k*_*a*_	m/(s · Pa)	Advection transfer coefficient of soil
*k*_*d*,*bm*_	m/s	Diffusion transfer coefficient of BM
*k*_*d*,*s*_	m/s	Diffusion transfer coefficient of soil
*q*_*ij*_	m^3^/s	Air current from compartment *i* to compartment *j*
*S*_*bm*_	m^2^	Indoor surface area of radon containing BM
*S*_*g*_	m^2^	Building area towards ground
*t*_*w*_	dimensionless	Radon transfer efficiency of water supply
*U*_*w*_	m^3^/s	Use rate of water
V	m^3^	Volume of the indoor
*ΔP*_*s* − *i*_	Pa	Soil-indoor pressure difference
λ	1/s	Radon decay constant
*λ*_*v*_	1/s	Ventilation rate

## Review

Most of the references reviewed in this article developed models for evaluating indoor radon concentrations based on the following mass balance equation:

$$ Indoor\kern0.5em  Radon\kern0.5em  Accumulation= Radon\kern0.5em  Entry\pm Radon\kern0.5em  Exchange- Decay\kern0.5em \mathrm{R}\mathrm{e} action $$

According to the above, changes in indoor radon concentrations are affected by the sources of radon entry, building ventilation (radon exchange), and decay reaction. First, we introduce a model for assessing indoor radon concentrations attributed to the three main sources of radon entry (soil, BM, and water) and decay reaction.

### Radon entry and decay reaction

#### Soil

First, we will consider a single-room building. As mentioned above, indoor radon concentrations are mainly affected by radon in the soil [4]. Accordingly, the change in indoor radon concentration for an infinitesimal time can be described as

1 $$ \frac{F_s{S}_g}{V}-\lambda {C}_i, $$

where the first and the second terms describe changes caused by radon in the soil and radioactive decay, respectively (see [5, 6, 9, 10, 14, 15] for more details). The total radon flux from the soil into the building *F*_*s*_ in Equation (abbre. Eq.) (1) can be calculated as

2 $$ {F}_s={k}_{d,s}\left({C}_s-{C}_i\right)+{k}_a\varDelta {P}_{s-i}{C}_s, $$

where the first and the second terms in the right-hand side of Eq. (2) describe diffusive and advective flow, respectively. Substituting Eq. (2) into Eq. (1), we obtain the following equation:

3 $$ \left\{{k}_{d,s}\left({C}_s-{C}_i\right)+{k}_a\varDelta {P}_{s-i}{C}_s\right\}\frac{S_g}{V}-\lambda {C}_i. $$

Although radon from groundwater contributes very little to indoor radon, it can constitute an important source in specific instances; the total radon flux from the ground, considering both radon in soil and groundwater, can be found in Ramola et al. [9]. In the meantime, Font [5] and Font and Baixeras [6] suggested evaluating total radon flux from the soil into the building *F*_*s*_ differently: the soil can be divided into two compartments, disturbed soil (DS, the volume of soil underneath a building from which radon can reach the basement of the building by diffusion and pressure driven flow) and undisturbed soil (US, the soil attached to the DS that is not influenced by the presence of the building). The radon concentrations in the US and in the DS are described in [5, 6]. However, for simplicity, throughout this article we develop a model using a total radon flux into the building that consider only DS, namely *F*_*s*_ in Eq. (2).

#### Building material

Next, we consider BM which is second leading source of radon into a building after soil. In some instances, BM can be the greater source of radon entry than the soil, such as in high-rise buildings [11, 12]. As illustrated in Fig. 1, radon entry from BM occurs through diffusion, as the difference in indoor and BM radon concentrations. Thus, the change in indoor radon concentration stemming from BM for an infinitesimal time can be described as

4 $$ {k}_{d,bm}\left({C}_{bm}-{C}_i\right)\frac{S_{bm}}{V} $$

(see [5, 6, 8, 10–12] for more details).

#### Water for living

Indoor radon concentrations can also depend on the amount of radon in a building’s water supply. In general, the influence of radon from a water supply on indoor radon concentrations is low; however, extremely high concentrations of radon in a water supply, when present, must be taken into consideration. To do so, changes in indoor radon concentrations caused by radon in a building’s water supply for an infinitesimal time can be taken as

5 $$ \frac{C_w{U}_w{t}_w}{V} $$

(see [5, 6, 13] for more details).

Then, from Eqs. (3), (4) and (5), the rate of change in indoor radon concentrations attributed to all sources of radon entry and decay can be represented by

6 $$ \frac{d{C}_i}{dt}=\left\{{k}_{d,s}\left({C}_s-{C}_i\right)+{k}_a\varDelta {P}_{s-i}{C}_s\right\}\frac{S_g}{V}+{k}_{d,bm}\left({C}_{bm}-{C}_i\right)\frac{S_{bm}}{V}+\frac{C_w{U}_w{t}_w}{V}-\lambda {C}_i, $$

where *dC*_*i*_ = *C*_*i*_(*t* + *dt*) − *C*_*i*_(*t*) and *dt* denotes an infinitesimal time.

### Radon exchange

#### Ventilation between indoor and outdoor air

As stipulated in the mass balance equation, radon exchange caused by ventilation and differences in indoor and outdoor radon concentrations must be considered. In general, indoor radon concentrations are higher in the winter season, compared to those in other seasons. This phenomenon is likely to be primarily related with ventilation. In considering radon exchange between indoor and outdoor air, we expand the model for assessing indoor radon concentration in Eq. (6) as

7 $$ \frac{d{C}_i}{dt}=\left\{{k}_{d,s}\left({C}_s-{C}_i\right)+{k}_a\varDelta {P}_{s-i}{C}_s\right\}\frac{S_g}{V}+{k}_{d,bm}\left({C}_{bm}-{C}_i\right)\frac{S_{bm}}{V}+\frac{C_w{U}_w{t}_w}{V}-{\lambda}_v\left({C}_i-{C}_o\right)-\lambda {C}_i, $$

where *λ*_*v*_(*C*_*i*_ − *C*_*o*_) describes the radon exchange between indoor and outdoor air [5, 6, 9–16]. In Eq. (7), one may assume that *λ* = 0 because *λ* is relatively smaller than *λ*_*v*_.

#### Ventilation among the compartments

Additionally, we must treat a building as a collection of different *N* compartments for more realistic models. In this case, from Eq. (7) the model for indoor radon concentration of *i* th compartment is derived as

8 $$ \frac{d{C}_i}{dt}=\left\{{k}_{d,s}\left({C}_s-{C}_i\right)+{k}_a\varDelta {P}_{s-i}{C}_s\right\}\frac{S_g}{V}+{k}_{d,bm}\left({C}_{bm}-{C}_i\right)\frac{S_{bm}}{V}+\frac{C_w{U}_w{t}_w}{V}-{\lambda}_v\left({C}_i-{C}_o\right)-\lambda {C}_i-{\displaystyle \sum_{\begin{array}{c}\kern1em j=1\kern1em \\ {}\kern1em j\ne i\kern1em \end{array}}^N}\left({q}_{ij}{C}_i-{q}_{ji}{C}_j\right)\frac{1}{V}, $$

where the last term describes the radon exchange between compartments *i* and *j* [5, 6].

When developing a model for assessing indoor radon concentrations of an actual building, some sources of radon entry and radon exchange may be removed from or added to Eq. (8) in accordance with the surroundings and the characteristics of the building. When adapting the model to an actual building, parameters other than the indoor radon concentration can either be measured or obtained from published data or certain time variation functions. To estimate indoor radon concentrations, one may seek to apply the following methods: obtaining the explicit solution of the differential equation at a steady state and using a proper numerical method that is usually based on a finite difference or finite element solutions to the equations. The models referenced in this review are summarized in Table 2.

**Table 2:** Summary of references reviewed in this article

Author(s)	Year	Factor				Application	Method^a^
		Soil	BM	Water	Vent.		
Kusuda et al. [16]	1980	-	-	-	○	Sample calculations	N.S.
Capra et al. [14]	1994	-	-	-	○	An environmental chamber	N.S.
Ramola et al. [9]	2011	○	-	-	○	Budhakedar area of Garhwal Himalaya in summer and winter	N.S.S.
Man and Yeung [11]	1999	-	○	-	○	Newly constructed, uninhabited high-rise buildings in Hong Kong	N.S.S.
Shaikh et al. [12]	2003	-	○	-	○	A multi-storey building in Mumbai, India over four seasons	N.S.S.
Vogiannis and Nikolopoulos [13]	2008	-	-	○	○	Thermal spas in Greece	N.M.
Jelle [15]	2012	○	○	-	○	Sample calculations	N.S.S.
Arvela et al. [10]	1988	○	○	-	○	Finnish dwellings in summer and winter	N.S.S.
Font [5]	1997	○	○	○	○	Mediterranean climate house and Swedish house^b^	N.M.
Font and Baixeras [6]	2003						

^a^N.M., N.S., and N.S.S. mean the numerical method using specially developed computer codes, the numerical solution of the differential equation, and the numerical solution of the differential equation at a steady state, respectively

^b^These applications were discussed in [7, 8]

## Conclusions

Mathematical models for estimating indoor radon concentrations provide not only a method of assessment but also an understanding between parameters that govern indoor radon levels. In order to estimate an individual’s cumulative indoor radon exposure, assessing indoor radon concentrations is necessary. To do so, one should seek to understand the mechanisms of radon entry and exchange in relation to side-specific surroundings and characteristics of a building. Accordingly, resultant models in consideration of said characteristics may be simpler than Eq. (8) or may necessitate additional research on factors of indoor radon concentrations (e.g., age of the BM and relative humidity).

Most of the models reviewed in this article utilized solutions at a steady state and considered parameters to be constant in order to estimate indoor radon concentrations. Such approaches are often adequate, as a steady state is rarely attained in actual buildings because of time-dependent factors (e.g., air exchange rates, which change with atmospheric conditions). For this reason, time-dependent solutions may be more accurate than steady state solutions in assessment of actual building conditions. Additionally, all of the models included in this review considered only deterministic models. However, a stochastic model designed to account for unrecognized factors and noise-corrupted measurements may offer better approximation of indoor radon levels.
